# Long Non-Coding RNAs and Related Molecular Pathways in the Pathogenesis of Epilepsy

**DOI:** 10.3390/ijms20194898

**Published:** 2019-10-02

**Authors:** Chiara Villa, Marialuisa Lavitrano, Romina Combi

**Affiliations:** School of Medicine and Surgery, University of Milano-Bicocca, via Cadore 48, 20900 Monza, Italy; marialuisa.lavitrano@unimib.it (M.L.); romina.combi@unimib.it (R.C.)

**Keywords:** long non-coding RNAs, epilepsy, epigenetics, gene

## Abstract

Epilepsy represents one of the most common neurological disorders characterized by abnormal electrical activity in the central nervous system (CNS). Recurrent seizures are the cardinal clinical manifestation. Although it has been reported that the underlying pathological processes include inflammation, changes in synaptic strength, apoptosis, and ion channels dysfunction, currently the pathogenesis of epilepsy is not yet completely understood. Long non-coding RNAs (lncRNAs), a class of long transcripts without protein-coding capacity, have emerged as regulatory molecules that are involved in a wide variety of biological processes. A growing number of studies reported that lncRNAs participate in the regulation of pathological processes of epilepsy and they are dysregulated during epileptogenesis. Moreover, an aberrant expression of lncRNAs linked to epilepsy has been observed both in patients and in animal models. In this review, we summarize latest advances concerning the mechanisms of action and the involvement of the most dysregulated lncRNAs in epilepsy. However, the functional roles of lncRNAs in the disease pathogenesis are still to be explored and we are only at the beginning. Additional studies are needed for the complete understanding of the underlying mechanisms and they would result in the use of lncRNAs as diagnostic biomarkers and novel therapeutic targets.

## 1. Introduction

Recent advances in genome-wide transcriptome sequencing technologies, along with the availability of suitable bioinformatics tools, have revealed that protein-coding genes only represent about 2% of the human transcripts, whereas the rest of the genome encodes for mainly non-coding RNAs (ncRNAs). Based on the length of the transcript, ncRNAs are commonly divided into two major groups: small (sncRNAs) and long non-coding RNAs (lncRNAs), typically larger than 200 nucleotides (nt). 

LncRNAs are transcribed by RNA polymerase II or III and have neither an appreciable open reading frame (ORF) nor a Kozak consensus sequence. Similar to messenger RNAs (mRNAs), they generally undergo maturation processes, including splicing, 5′ capping, and 3′ polyadenylation [1]. The LncRNA gene structure is also similar to the one of protein-coding genes, displaying both introns and exons, even though the co-transcriptional splicing process is less efficient [2]. Although, by definition, lncRNAs are not widely translated, increasing evidences have revealed that some lncRNAs can be translated into peptides and potentially even functional proteins, bearing biological functions [3]. Recent ribosome profiling studies reported that some lncRNAs bind to ribosomes and contain either short ORFs or Internal Ribosomal Entry Sites (IRES) and other translation elements, which increases the possibility that their coding potential has been largely underestimated [4]. Concerning cellular localization, the lncRNA mature molecules are found within the nucleus, the cytosol, and even in mitochondria [5,6]. According to their position in the genome and orientation in respect to the adjacent protein-coding genes, lncRNAs can be subclassified into major groups: sense, antisense, intronic, intergenic, and bidirectional [7]. Their functions seem to be linked to the genome location. Increasing evidences indicated that lncRNAs play a key role as regulator molecules to control nuclear organization, RNA processing, and transcriptional and post-transcriptional modulation of gene expression [8]. They might act as competing endogenous RNAs (ceRNAs) regulating other RNA transcripts by competitively inhibiting microRNAs (miRNAs), a class of conserved sncRNAs (18 to 25 nucleotides) [9]. Moreover, they are involved in a wide variety of biological processes, including cell death, proliferation, differentiation, chromatin remodeling, genomic imprinting, X-chromosome inactivation, immune response, and organogenesis [10]. LncRNAs have been also implicated in the onset of several human diseases, such as cancer, autoimmune, metabolic, cardiovascular, and neurological disorders [11]. 

LncRNAs display distinctive evolutionarily conserved and tissue-specific patterns [12], with a large fraction of them (approximately 40% of all lncRNAs) being expressed in the brain, often showing precise spatial-temporal control of gene expression during the development [13,14]. In the central nervous system (CNS), some of the lncRNAs play possible roles in neuronal function, CNS development and maintenance of memory, cognitive function and synaptic plasticity [15]. Given their specific expression in the nervous system and the functions in the normal cellular processes, a dysregulation or an abnormal expression of lncRNAs might cause an impairment of brain function and development, which leads to several neurodevelopmental and neurodegenerative disorders, including epilepsy, autism spectrum disorders (ASDs), and Alzheimer’s disease (AD) [16,17]. 

Epilepsy is a group of neurological and chronic disorders of the brain with a high prevalence in the population and affecting people of whole range of ages [18]. The presence of recurrent and unprovoked seizures, representing a major factor of worse quality of life and increasing the risk for morbidity, is a common feature of the epileptic conditions. Epilepsy onset has been attributed to an imbalance of inhibitory and excitatory neurons in the CNS, which leads to abnormal hypersynchronous electrical activity of neuronal networks [19]. However, cellular and molecular mechanisms responsible for epileptogenesis are not completely understood [20]. In the etiology of most epilepsies, a combination of genetic and acquired factors is involved, while predominantly genetic epilepsies only constitute a minority of all seizure disorders [21]. 

Emerging evidences suggested an important role of lncRNAs in molecular mechanisms that are already associated with epilepsy, mainly regarding brain excitability and seizure thresholds. In particular, they are known to regulate several processes: e.g., gliosis, neuroinflammation, neuronal death, synaptic plasticity, changes to ion channels and transmitter receptors, and network-level remodeling [22]. 

## 2. Expression Profiling Studies Firstly Suggested the Role of lncRNA in Epilepsy

The involvement of lncRNAs in the pathogenesis of epilepsy was firstly suggested by studies that reported their aberrant expression both in brain specimen from epileptic patients who underwent surgery and in experimental animal models of epilepsy [23,24,25]. Among these reports, the first study explored the role of lncRNAs by comparing (using microarray analysis) their expression in pilocarpine or kainic acid (KA)-induced epilepsy models (two different animal models of chronic epilepsy commonly used to elucidate epilepsy pathogenesis) versus the control mice. The authors found 384 differentially expressed lncRNAs in the pilocarpine model and 279 in the KA-induced one when comparing the nervous tissue from controls with that of treated mice. Among them, 54 and 14 lncRNAs, respectively, are close to protein-coding genes and seem to induce significant changes in gene expression, which suggests their putative positive regulation. A pathway analysis indicated their involvement in neuron differentiation, embryonic appendage morphogenesis and inflammation in chronic epilepsy [23]. Interestingly, recent studies reported that epigenetic mechanisms are not only able to regulate the expression of protein-coding genes, but also affect lncRNAs, observing that wide methylation changes occur more frequently in the promoters of ncRNAs than those of the protein-coding genes [26]. In this regard, Xiao et al. [24] identified an altered methylation profile of lncRNAs that are associated with the development and progression of temporal lobe epilepsy (TLE), the most common form of adult epilepsy and the hardest to treat, often evolving in drug resistance. In particular, they found that these aberrant methylated lncRNAs are related to specific biological processes that are frequently involved in the epileptogenesis, such as ion/gated channel activity, synaptic transmission or γ-aminobutyric acid (GABA) receptor activity [24]. A further study has been performed attempting to evaluate dysregulated lncRNAs with corresponding mRNAs in the pilocarpine mouse model in both the cortex and the hippocampus, which are the most important brain regions damaged by TLE [25]. The authors found several differentially expressed lncRNAs (mostly intergenic) and mRNAs, which are unique for each brain region. Gene ontology (GO) and pathway analysis reported that the dysregulated mRNAs were closely related to well-known epileptogenic biological processes: neuronal differentiation, inflammation, calcium ion regulation, and extracellular matrix remodeling [25]. Moreover, the analysis of protein-protein interaction revealed that dysregulated protein-coding transcripts were interconnected around the mammalian target of rapamycin (mTOR) and RE-1 silencing transcription factor (REST) pathways, which are involved in cellular signaling transduction and epigenetic mechanisms during the hippocampal sclerosis, respectively [27,28]. No common genes with altered expression were detectable by comparing the results in the pilocarpine model brain from the two above reported studies [23,25].

## 3. LncRNAs Associated with Epilepsy and Involved in Synaptic Plasticity

During the development, neurons extend axons and dendrites to form connections and allow for synapse formation (synaptogenesis). These processes involve complex regulations on gene expression and signal transduction. Neuronal cells can modify their synaptic connections in response to increases or decreases in their activity. This biological process, which is known as synaptic plasticity, is essential for maintaining the stability of the nerve pathway and representing the basis of memory, learning and cognition. Emerging evidences indicated that many lncRNAs are actively involved in the regulation of synaptic plasticity, and thus the fidelity of memory and cognitive processes, by integrating and dynamically monitoring multiple transcriptional and post-transcriptional events [29,30]. Moreover, lncRNAs do not only regulate the expression of genes that are involved in neurite outgrowth, but they are also able to modulate ion channel stoichiometry, thus altering the synaptic connectivity and the excitatory properties of neurons. 

### 3.1. Brain Cytoplasmic 1 RNA (BC1)

The rodent-specific BC1 and the non-homologous primate-specific BC200 lncRNAs are thought to modulate local protein synthesis in post-synaptic dendritic microdomains and maintain long-term plasticity [31]. BC1 is primarily expressed in the dendrites and somata of neurons, which are enriched at the hippocampus, the olfactory bulb, and the cortex [32]. In neurons, BC1 operates as a translational repressor targeting eukaryotic initiation factor 4A (eIF4A), an adenosine triphosphate (ATP)-dependent RNA helicase. The BC1-eIF4A interaction results in decoupling ATP hydrolysis from RNA duplex unwinding, modulating the synthesis of synapse-related proteins and thus preserving the long-term plasticity [33]. Moreover, like its homologous BC200, BC1 interacts with other RNA-binding proteins, such as fragile X mental retardation protein (FMRP) and poly(A)-binding protein (PABP), at the level of post-synaptic dendritic microdomains [31,34]. 

Some studies performed using different in vivo models have investigated the role of BC1 in the pathogenesis of epilepsy. In BC1-null mice, some of the authors reported that this lncRNA is required in the maintenance of the excitation–repression homeostasis at the synapse, which was probably due to hyperactive group I metabotropic glutamate receptors (mGluRs)-triggered translation: its loss results in increased neuronal excitability, reducing convulsive thresholds [35]. These data were supported by a following study, in which the BC1 RNA levels have been found to be significantly decreased in the hippocampus of Wistar audiogenic rats (WARs), a genetically selected strain of rat predisposed to sound-induced seizures, useful as model for behavioral and neuropathological features that were observed in human TLE [36]. In a pilocarpine-induced rat model, other authors investigated the dynamic expression of BC1 and its interaction with eIF4A in the hippocampus after status epilepticus (SE). They found that the levels of this lncRNA vary at different time points (3d, 1w, and 2w post-SE) and that the BC1-eIF4A interaction occurs after the SE, which strengthens the involvement of BC1 in the synaptic connections and abnormal formation of dendrites after seizures [37].

### 3.2. Brain-Derived Neurotrophic Factor Antisense RNA (BDNF-AS)

BDNF antisense RNA (BDNF-AS, also annotated as BDNF-OS) is a lncRNA that is transcribed from the opposite strand of brain-derived neurotrophic factor (BDNF), a neurotrophin that is involved in neuronal differentiation, growth, maturation, synaptic plasticity, learning, and memory processes. BDNF mRNA and protein are both markedly upregulated by seizure activity in the hippocampus of animal models of epilepsy as well as in human brain tissue displaying increased epileptic activities [38]. BDNF and BDNF-AS form double-stranded duplexes, which suggests a potential role for BDNF-AS in the post-transcriptional regulation of BDNF. In fact, in vitro cell studies showed that BDNF-AS can negatively regulate BDNF expression [39]. Concerning epilepsy, a study found that the expression of BDNF is upregulated in human neocortex removed as a treatment of intractable seizures, whereas the levels of BDNF-AS are significantly decreased [40]. These data suggest that the mRNA-lncRNA interaction might represent a regulatory network of human brain plasticity and that inhibiting BDNF pathway could be used as a potential therapeutic strategy for epilepsy. 

### 3.3. SCN1ANAT: Natural Antisense Transcript (NAT) of Sodium Voltage-Gated Channel α-1 Subunit (SCN1A)

Loss or gain-of-function mutations of *SCN1A*, encoding for the α subunit of the voltage-gated sodium channel Nav1.1, are both associated with a spectrum of seizure-related disorders in humans, which ranges from a relatively milder form of febrile seizures to a more severe epileptic condition known as Dravet syndrome (DS), previously named severe myoclonic epilepsy of infancy (SMEI). Concerning DS, the majority of mutations are loss-of-function and result in *SCN1A* haploinsufficiency [41]. Consequently, the possibility to directly upregulate the remaining normal copy of the gene might be a way to attenuate disease manifestations, thus representing a potential therapeutic strategy. Another way is represented by the specific silencing of the well-known *SCN1A* natural antisense transcript (NAT), named SCN1ANAT, which is transcribed by the opposite strand of *SCN1A* gene and that normally modulates the expression of the *SCN1A* mRNA. In this frame, Hsiao and colleagues [42] upregulated specifically *SCN1A* expression in a DS mouse model by using AntagoNATs (oligonucleotide-like compounds) blocking *SCN1ANAT*, while not altering >90% of all of the expressed genes, including other highly homologous sodium channel subunits and genes that are immediately adjacent to *SCN1A* on the chromosome [42]. This study demonstrated that the upregulation of the haploinsufficient gene expression in the brain leads to a significant improvement in the excitability of hippocampal interneurons and seizure phenotype [42]. Clinical trials phase II using AntagoSCN1ANAT have been already announced by several companies. In particular, the first program is the OPK88001 (previously CUR-1916) by OPKO Health whose starting was scheduled in 2019. A second program was announced by Stoke Therapeutics, which was also developing an antisense oligonucleotide treatment to boost expression of SCN1A. This oligonucleotide binds to a form of mRNA and leads to an increase in the levels of mature mRNA and Nav1.1 protein. The company shared preliminary data with efficacy in a mouse model and planned to initiate clinical trials in 2020. 

## 4. LncRNAs Associated with Epilepsy and Involved in Neuron Apoptosis

Apoptosis is a form of programmed cell death that is essential for the survival and development of living organisms, controlling cell activity, differentiation, and proliferation. Bcl-2 family protein and caspase, including both pro-apoptotic (such as Bax) and anti-apoptotic members (such as Bcl-2), play an essential role as modulators of the apoptotic response. Apoptosis represents one of the key molecular mechanisms underlying cell death following SE and alterations in apoptosis-associated signaling pathways have been largely reported in both TLE tissue and animal TLE models [43]. Moreover, decreased levels of Bcl-2 and increased levels of Bax cleaved caspase-3 were observed in apoptotic neurons in the hippocampus of a KA-induced epilepsy model [44]. Emerging evidences also revealed a role of lncRNAs in regulating neuron apoptosis, mainly by acting as ceRNA to target miRNAs. 

### 4.1. H19

The RNA polymerase II transcribes H19, the first identified lncRNA, from *H19* gene mapped on chromosome 11 and it is normally subject to post-transcriptional RNA maturation process. It is a paternally imprinted non-coding transcript only expressed from the maternal allele and it shows a highly conserved secondary structure. *H19* is highly expressed during fetal life and downregulated after birth, except for persistent expression in the adult heart and skeletal muscle. It plays different functions depending on physiological and pathological state. In the CNS, *H19* is overexpressed in glioblastoma tissues and it promotes the invasion, angiogenesis, proliferation, and migration of glioblastoma cells [45]. A possible involvement of this lncRNA in epilepsy has only recently been explored. Using genome-wide expression profiling and bioinformatic analyses in order to reveal the functions and regulatory mechanisms of H19, a study detected a large number of genes that were differentially expressed when H19 was either overexpressed or knocked down in a KA-induced epileptic rat model [46]. Pathway and functional analyses suggested its possible involvement in a wide spectrum of epileptogenic processes, including cell proliferation, apoptosis, demyelination, mitogen-activated protein kinase (MAPK) activation, inflammatory, and immune responses [46]. In another study by the same group, H19 resulted the most markedly differentially expressed lncRNA in the hippocampus of epileptic rat models of TLE [47]. In particular, it resulted in being downregulated at the acute period of seizure due to a probable inhibition that was caused by cerebral hypoxia, while upregulated in the seizure-free period of TLE. The authors suggested that, during the chronic period, repeated and spontaneous recurrent seizures induce hypoxia-reoxygenation, which in turn leads to H19 altered levels and finally to exacerbation of the hippocampal damage [47].

The same authors also reported that, in the hippocampus of TLE rat model, H19 promotes neuron apoptosis by acting as ceRNA to sponge miRNA let-7b in the regulation of Casp3 expression [47]. Finally, they showed that an overexpression of H19 resulted in the activation of microglia and astrocytes via the modulation of the JAK/STAT pathway and stimulated the release of proinflammatory cytokines in the hippocampus, which supports its role in inflammatory response, as previously suggested [46,48].

### 4.2. FTX

FTX (five prime to Xist) is a kind of evolutionarily conserved lncRNA, which regulates Xist expression and chromatin structure within the X-inactivation center region [49]. This lncRNA is largely dysregulated during tumorigenesis, even though its function in different types of cancer is contradictory. For instance, FTX is upregulated in gliomas and it facilitates the growth and metastasis through sponging miR-342-3p [50]. Conversely, in hepatocellular carcinoma (HCC), it acts as tumor suppressor, inhibiting proliferation and invasion by binding miR-374a [51]. Other authors reported that FTX is significantly downregulated in ischemic areas and it regulates the apoptosis cardiomyocytes by targeting miR-29b-1-5p and Bcl2l2 [52].

Concerning its role in the epilepsy pathogenesis, only one study has been performed in a pilocarpine-induced rat model up to now. The authors found that FTX expression is significantly reduced in the hippocampal tissue of SE rats, which suggests that the cerebral hypoxia induced by seizures might inhibit FTX expression. Moreover, they showed that the upregulation of FTX ameliorates seizures, shortening their latency, and inhibits the hippocampal neuron apoptosis. Investigating the functional target of FTX, the same authors found that this lncRNA negatively regulates miR-21-5p expression during epileptogenesis by targeting its 3′UTR. The upregulation of this miRNA attenuates the anti-apoptosis ability of FTX through the regulation of sex-determining region Y-box 7 (SOX7) expression, a transcription factor that is involved in several developmental processes, including apoptosis. Collectively, these findings demonstrated a novel FTX-mediated mechanism in SE-induced neural damage through miR-21-5p/SOX7 axis in the regulation of cellular apoptosis, which might provide new targets in the development of these lncRNA-based therapeutic strategies [53].

### 4.3. Urothelial Cancer Associated 1 (UCA1)

UCA1, which was firstly identified in bladder cancer, is also significantly upregulated in many types of carcinomas, such as hepatocellular, breast, colorectal, and gastric cancer, allowing it to be considered as a universal oncogenic lncRNA. In the CNS, some authors found that it regulates stem cell proliferation and differentiation to astrocytes and neurons [54]. Concerning its potential involvement in epilepsy, UCA1 resulted abnormally methylated in the hippocampus of TLE patients after surgical resection, although the SE underlying mechanism was not studied [55]. Wang and collaborators, going deep inside into the role of this lncRNA in the pathogenesis of epilepsy, showed that UCA1 is able to induce or even aggravate epilepsy in a pilocarpine-induced rat model through the interaction with NF-kB and that UCA1 expression in the peripheral blood of epilepsy rats is positively correlated with that in brain tissue [56]. In particular, studying brain tissues in temporal hippocampus of rats through expression profiling, they found higher expression levels of both genes in epileptic rats than in the control group. Moreover, a higher level of UCA1 mRNA was also detected in peripheral blood of the experimental group than in the control group. The authors concluded that the expressions of NF-Kb and UCA1 are in the dynamic change in the formation of epilepsy, which suggests a role of UCA1 in the pathogenesis of the disease [56].

On the contrary, other reports are available suggesting a benefit side of UCA1, in which it suppressed pilocarpine-induced epilepsy by inhibiting the apoptosis of hippocampal neurons through the regulation of miR-495 and nuclear factor erythroid 2-related factor2 (Nrf2) bound to the antioxidant response element (ARE) pathway, found to protect the brain from damage induced by epileptic seizures [57,58]. In particular, Geng and colleagues [56] found that Nrf2 was negatively regulated by miR-495 and that lncRNA UCA1 was down-regulated and miR-495 was up-regulated in pilocarpine-induced epilepsy rat models. The anticonvulsant property of the activation of Nrf2-ARE signal pathway and its protection effect on cognitive deficits induced by epileptic seizures was also previously reported [57].

## 5. LncRNAs Associated with Epilepsy and Involved in Both Synaptic Plasticity and Apoptosis

### 5.1. Metastasis-Associated Lung Adenocarcinoma Transcript 1 (MALAT1)

MALAT1, which is also known as nuclear-enriched abundant transcript 2 (NEAT2), is one of the most widely studied lncRNAs and largely conserved in mammals. It is located on chromosome 11q13 and firstly identified in a screen for transcripts associated with metastasis and patient survival in non-small cell lung cancer. This lncRNA is ubiquitously expressed in almost all human tissues and it is very abundant in brain, especially in the high-activities areas of the human neocortex. MALAT1 regulates many genes that are involved in dendritic and synapse development, exerting its molecular functions through alternative splicing and transcriptional and post-transcriptional regulation of them [59]. Its depletion in cultured hippocampal neurons resulted in a significant decrease in synaptic density, whereas its upregulation led to an increase of synaptogenesis [60] and many neuropsychiatric disorders (e.g., ASDs and schizophrenia) showed compromised synaptic function [61]. 

Regarding its role in epilepsy, some authors recently found that a downregulation of MALAT1 is able to inhibit excessive autophagy and the apoptosis of hippocampal neurons in a pilocarpine-induced epilepsy rat model by the activation of the PI3K/Akt signaling pathway [62]. The authors demonstrated that MALAT1 was significantly augmented in the hippocampus of rats with epilepsy and that the injection of an antisense molecule (si-MALAT1) was efficient in reducing this level resulting in a more prolonged latency duration of epilepsy seizure. The authors proposed a model where the high expression of MALAT1 promotes apoptosis by inhibiting the PI3k/Akt signaling pathway that was previously reported to modulate cell survival after oxidative stress events that were provoked by epileptic seizures [63].

Although MALAT1 could be a potential target of therapy, it might be very complicated to specifically target MALAT1 in pathological conditions by simply overexpressing or silencing it, owing to the fact that it is ubiquitously expressed and it is also involved in normal physiological functions (e.g., synapse formation, vascular growth, and skeletal myogenesis). 

### 5.2. Plasmacytoma Variant Translocation 1 (PVT1)

PVT1 is a lncRNA with oncogenic function in multiple cancers and it maps on chromosome 8q24, a recognized cancer risk region that is shared with the well-known MYC oncogene. An increasing number of studies reported that this lncRNA is involved in proliferation, migration, and invasion of cancer cells. Moreover, PVT1 is upregulated in different carcinomas and its overexpression is associated with reduced survival in patients [64]. 

Its possible role in the pathogenesis of epilepsy has only recently been proposed by a single study that was performed in a pilocarpine-induced rat model in which the authors found an increased expression of PVT1 in the hippocampus [65]. Moreover, the silencing of this lncRNA decreased neuronal loss and the expression of cleaved caspase-3 and Bax, while increased pro-caspase-3 and Bcl2 in hippocampus tissues of epileptic rats, suggesting its role in neuron apoptosis. Finally, the silencing of PVT1 also causes an increase in the expression of BNDF, which is known to be involved in the survival of neuronal populations during development, synaptogenesis, dendritic, and axonal growth [65].

## 6. LncRNAs Associated with Epilepsy and Involved in Neuronal Differentiation

LncRNAs play an important role also in the development of nervous system, a series of events occurring during the embryonic and adult brains throughout life leading to a neuronal cell fate determination due to their characteristic differential and tissue-specific expression patterns. By analyzing the expression of lncRNAs in different kinds of neural cells, it has been found their potential involvement in neuronal-glia fate switching, lineage specification and oligodendrocyte maturation [66]. Moreover, another study revealed that lncRNAs are associated with protein-coding genes in neuronal development and they are important in maintaining the intrinsic morphology and the characteristics of neurons were reported [67]. 

### 6.1. Evf2

Evf2, also known as Dlx6as or Dlx6os1, is a lncRNA that is transcribed from the ultra-conserved intergenic region between *Dlx5* and *Dlx6* genes, encoding two members of the DLX homeodomain-containing protein family, required for interneuron [68]. In the developing mouse forebrain, the Evf2 regulated transcription factors are critical in the development of interneurons producing GABA, the major inhibitory neurotransmitter, and its loss resulted in defects in the GABAergic system [69]. Indeed, alterations in GABA signaling have been linked to epilepsy, autism, schizophrenia, and other neurological disorders. A recent study, which was performed in a mouse model, demonstrated that this lncRNA decreases seizure susceptibility and mice lacking Evf2 were susceptible to more severe and frequently seizures, due to impaired GABA-dependent neuronal circuitry [70]. In particular, a decrease frequency of GABAergic synaptic transmission was observed in the cingulate cortex (CG1) deep layer (V) and electroencephalogram (EEG) recordings from the medial prefrontal cortex (mPFC) detected an increased seizure severity. These observations suggested that Evf2 regulatory events are critical in GABAergic synaptic transmission in these two different adult brain regions controlling seizure susceptibility in adult mice [70].

### 6.2. Nuclear-Enriched Abundant Transcript 1 (NEAT1)

Originally named as nuclear-enriched abundant transcript 1 and subsequently renamed to nuclear paraspeckle assembly transcript 1, NEAT1 represents one of the most abundant lncRNAs in the nucleus localizing to specific structures, called paraspeckle. Unlike other lncRNAs, which commonly lack sequence conservation, NEAT1 is relatively conserved across mammalian species, which supports its important biological function. This ubiquitous lncRNA is highly expressed in neurons and it was found to be upregulated during neuronal and glia differentiation [66]. A transcriptome analysis of human tissue demonstrated altered levels of NEAT1 in the CNS in major neurodegenerative disorders as well as in some disease models. As this lncRNA regulates cellular pathways that are commonly affected in neurodegenerative diseases, it was found to be dysregulated in AD, Parkinson’s disease (PD), amyotrophic lateral sclerosis (ALS), frontotemporal dementia (FTD), and Huntington’s disease (HD) [71]. 

Interestingly, the chromosomal 11q13 region containing *NEAT1* locus has been previously involved in idiopathic seizures [72]. Moreover, in a small cohort of TLE patients, it was found to be upregulated in the cerebral cortex, specifically in high activity as compared to low activity regions [40,73]. However, NEAT1 is transiently downregulated in response to acute activity in pilocarpine or KA-induced epilepsy rat models. The same authors also reported that this lncRNA contributes to the modulatory capacity of neuronal excitability, binding ion channel modulatory proteins, such as the potassium channel-interacting protein KCNAB2, which is itself related to epilepsy and neuronal excitability [73].

## 7. Conclusions

**Figure 1:**
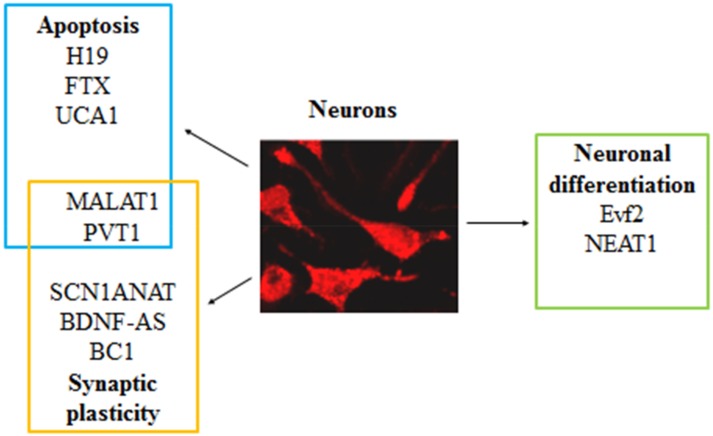
A summary of the most dysregulated lncRNAs in epilepsy. Each lncRNA is reported together with the biological process in which it has been implicated.

Whole-transcriptome analyses have provided important insights into the clinical and biological relevance of lncRNAs in epilepsy as emerging crucial regulators of several processes that occur during epileptogenesis (Table 1). However, how these lncRNAs are involved in this disorder is starting to be unraveled and further research needs to be done. Figure 1 shows that these lncRNAs are, in fact, involved in some pathways previously associated with epilepsy (Figure 1). With a deeper understanding of the molecular mechanisms by which they act, effective and novel targets will be identified for the development of therapeutic strategies. In this regard, a potential therapeutic intervention, based on the reduction of the UBE3A antisense transcript (UBE3A-ATS) lncRNA (using specific antisense oligonucleotides, ASOs), has been successful proposed in patients affected by Angelman syndrome, a genetic disorder that is caused by maternal deficiency of the imprinted gene *UBE3A* (encoding an E3 ubiquitin ligase) and characterized by intellectual disability, severe speech impairment, developmental delay, seizures, and ataxia [74]. A similar approach using ASO-mediated therapies has been also applied in some ongoing phase I/II clinical trials for the treatment of some types of cancers [75], ALS [76], and spinal muscular atrophy (SMA) [77]. Therefore, the experience gained in these clinical fields may also lead to their applications in epilepsy: promising results have already been achieved modulating SCN1A expression in vivo by specific ASO blocking SCN1ANAT lncRNA [47]. One limit of the studies on the correlation between epileptogenesis and lncRNAs is that the available data are mostly associated with TLE (patients and experimental models), while studies in other types of epilepsy are still lacking. Moreover, the results that were obtained from experimental models are not always parallel to those obtained in patients. Subsequently, it would be necessary to design more studies in patients to support the relevance of lncRNAs in epilepsy. Finally, an important limiting issue is that here reported studies describing changes in lncRNAs were obtained from patients with pharmacoresistant epilepsy. Thus, we could not exclude that observed changes are associated with the pharmacoresistance. Interestingly, a contribution of lncRNAs in pharmacoresistance to drugs is well reported in cancer [78]. Several studies in cancer cells demonstrated that lncRNAs are differently expressed in sensitive and resistant cells mostly positively correlating with chemoresistance. They modulate the efficacy of drugs via regulating gene expression either directly or indirectly. Moreover, they demonstrated that one lncRNA can regulate many drugs resistance, and conversely one drug resistance can be regulated by many lncRNAs [78].

Furthermore, it has been reported that lncRNAs can be detected in biological fluids, such as plasma or urine, and that they show a dynamic alteration upon diseases [79,80]. Due to their abundance and long-term stability in easily accessible body fluids, especially when included in apoptotic bodies or exosomes [81], lncRNAs may be considered as a novel class of non-invasive clinical diagnostic and prognostic biomarker. In support of this perspective, their use has already been successfully exploited in the field of oncology [82], where lncRNAs have been associated with the prognosis of patients that are affected by hepatocellular carcinoma and breast and colorectal cancers [83]. 

In conclusion, although many studies have been performed in both clinical patients and different disease models, the exact role and impact of lncRNAs in epileptogenesis is beginning to be explored, but it promises to open a new scenario for early diagnosis and setting-up of innovative therapies based on manipulations of their functions. Moreover, the use of lncRNAs as prognostic biomarkers should be supported with studies in patients or animals, showing lncRNAs before the expression of epileptic activity (e.g., subjects with traumatic brain injury before the development of post-traumatic epilepsy). Hereby, further investigation into the mechanisms by which lncRNAs act will provide a deeper understanding of the onset, development, and pathogenesis of the disease. 

## Figures and Tables

**Table 1:** Epilepsy-associated lncRNAs in literature.

LncRNA	Model/Tissue, Species	Effects/Findings	References
BC1	BC1-null model, mouse	lowered seizure threshold	[35]
	WAR model, rat	decreased levels	[36]
	PILO model, rat	altered levels at different time points after SE	[37]
BDNF-AS	resected neocortex from TLE patients, human	decreased levels correlating with upregulated BDNF	[40]
SCN1ANAT	DS model, mouse	improved seizure phenotype through its specifically blocking	[42]
H19	KA model, rat	involved in a broad spectrum of epileptogenic processes	[46]
	resected hippocampus from TLE patients, human	upregulated in the latent period of TLE and contributed to apoptosis by inhibiting let-7b	[47]
	KA model, rat		
	resected hippocampus from TLE patients, human	involved in microglia activation modulating JAK/STAT signaling	[48]
	KA model, rat		
FTX	PILO model, rat	ameliorated seizure activity by inhibiting apoptosis	[53]
UCA1	resected hippocampus from TLE patients, human	abnormally methylated	[55]
	PILO model, rat	increased expression positively correlating with the nuclear transcription factor NF-kB	[56]
	PILO model, rat	suppressed epilepsy by inhibiting apoptosis	[58]
MALAT1	PILO model, rat	inhibited apoptosis and autophagy by its downregulation	[62]
PVT1	PILO model, rat	decreased neuronal loss and increased BDNF expression through its silencing	[65]
Evf2	knock-out model, mouse	increased susceptibility to more severe and frequently seizures	[70]
NEAT1	resected neocortex from TLE patients, human	upregulated in high activity regions	[40,73]
	PILO and KA models, rat	transiently downregulated in response to acute activity	[73]

WAR: Wistar audiogenic rat; PILO: pilocarpine; SE: status epilepticus; DS: Dravet syndrome; KA: kainic acid; TLE: temporal lobe epilepsy.

## References

[1] Qureshi I.A., Mattick J.S., Mehler M.F. (2010). Long non-coding RNAs in nervous system function and disease. Brain Res..

[2] Ulitsky I., Bartel D.P. (2013). LincRNAs: Genomics, evolution, and mechanisms. Cell.

[3] Pan J., Meng X., Jiang N., Jin X., Zhou C., Xu D., Gong Z. (2018). Insights into the Noncoding RNA-encoded Peptides. Protein Pept. Lett..

[4] Li J., Liu C. (2019). Coding or Noncoding, the Converging Concepts of RNAs. Front. Genet..

[5] Rackham O., Shearwood A.M., Mercer T.R., Davies S.M., Mattick J.S., Filipovska A. (2011). Long noncoding RNAs are generated from the mitochondrial genome and regulated by nuclear-encoded proteins. RNA.

[6] Van Heesch S., van Iterson M., Jacobi J., Boymans S., Essers P.B., de Bruijn E., Hao W., MacInnes A.W., Cuppen E., Simonis M. (2014). Extensive localization of long noncoding RNAs to the cytosol and mono- and polyribosomal complexes. Genome Biol..

[7] Quinn J.J., Chang H.Y. (2016). Unique features of long non-coding RNA biogenesis and function. Nat. Rev. Genet..

[8] Yao R.W., Wang Y., Chen L.L. (2019). Cellular functions of long noncoding RNAs. Nat. Cell. Biol..

[9] Cai Y., Wan J. (2018). Competing Endogenous RNA Regulations in Neurodegenerative Disorders: Current Challenges and Emerging Insights. Front. Mol. Neurosci..

[10] Fernandes J.C.R., Acuña S.M., Aoki J.I., Floeter-Winter L.M., Muxel S.M. (2019). Long Non-Coding RNAs in the Regulation of Gene Expression: Physiology and Disease. Noncoding RNA.

[11] Hu G., Niu F., Humburg B.A., Liao K., Bendi S., Callen S., Fox H.S., Buch S. (2018). Molecular mechanisms of long noncoding RNAs and their role in disease pathogenesis. Oncotarget.

[12] Vance K.W., Ponting C.P. (2014). Transcriptional regulatory functions of nuclear long noncoding RNAs. Trends Genet..

[13] Derrien T., Johnson R., Bussotti G., Tanzer A., Djebali S., Tilgner H., Guernec G., Martin D., Merkel A., Knowles D.G. (2012). The GENCODE v7 catalog of human long noncoding RNAs: Analysis of their gene structure, evolution, and expression. Genome Res..

[14] Ng S.Y., Lin L., Soh B.S., Stanton L.W. (2013). Long noncoding RNAs in development and disease of the central nervous system. Trends Genet..

[15] Wu P., Zuo X., Deng H., Liu X., Liu L., Ji A. (2013). Roles of long noncoding RNAs in brain development, functional diversification and neurodegenerative diseases. Brain Res. Bull..

[16] Wan P., Su W., Zhuo Y. (2017). The Role of Long Noncoding RNAs in Neurodegenerative Diseases. Mol. Neurobiol..

[17] Cortini F., Roma F., Villa C. (2019). Emerging roles of long non-coding RNAs in the pathogenesis of Alzheimer’s disease. Ageing Res. Rev..

[18] De Boer H.M., Mula M., Sander J.W. (2008). The global burden and stigma of epilepsy. Epilepsy Behav..

[19] Gong X.W., Li J.B., Lu Q.C., Liang P.J., Zhang P.M. (2014). Effective connectivity of hippocampal neural network and its alteration in Mg2+-free epilepsy model. PLoS ONE.

[20] Pitkänen A., Lukasiuk K. (2011). Mechanisms of epileptogenesis and potential treatment targets. Lancet Neurol..

[21] Steinlein O.K. (2008). Genetics and epilepsy. Dialogues Clin. Neurosci..

[22] Henshall D.C., Kobow K. (2015). Epigenetics and Epilepsy. Cold Spring Harb. Perspect. Med..

[23] Lee D.Y., Moon J., Lee S.T., Jung K.H., Park D.K., Yoo J.S., Sunwoo J.S., Byun J.I., Lim J.A., Kim T.J. (2015). Dysregulation of long non-coding RNAs in mouse models of localization-related epilepsy. Biochem. Biophys. Res. Commun..

[24] Xiao W., Cao Y., Long H., Luo Z., Li S., Deng N., Wang J., Lu X., Wang T., Ning S. (2018). Genome-Wide DNA Methylation Patterns Analysis of Noncoding RNAs in Temporal Lobe Epilepsy patients. Mol. Neurobiol..

[25] Jang Y., Moon J., Lee S.T., Jun J.S., Kim T.J., Lim J.A., Park B.S., Yu J.S., Park D.K., Yang A.R. (2018). Dysregulated long non-coding RNAs in the temporal lobe epilepsy mouse model. Seizure.

[26] Li Y., Zhang Y., Li S., Lu J., Chen J., Wang Y., Li Y., Xu J., Li X. (2015). Genome-wide methylome analysis reveals epigenetically dysregulated ncRNAs in human breast cancer. Sci. Rep..

[27] Sosunov A.A., Wu X., McGovern R.A., Coughlin D.G., Mikell C.B., Goodman R.R., McKhann G.M. (2012). The mTOR pathway is activated in glial cells in mesial temporal sclerosis. Epilepsia.

[28] Goldberg E.M., Coulter D.A. (2013). Mechanisms of epileptogenesis: A convergence on neural circuit dysfunction. Nat. Rev. Neurosci..

[29] Leal G., Comprido D., Duarte C.B. (2014). BDNF-induced local protein synthesis and synaptic plasticity. Neuropharmacology.

[30] Maag J.L., Panja D., Sporild I., Patil S., Kaczorowski D.C., Bramham C.R., Dinger M.E., Wibrand K. (2015). Dynamic expression of long noncoding RNAs and repeat elements in synaptic plasticity. Front. Neurosci..

[31] Muddashetty R., Khanam T., Kondrashov A., Bundman M., Iacoangeli A., Kremerskothen J., Duning K., Barnekow A., Huttenhofer A., Tiedge H. (2002). Poly(A)-binding protein is associated with neuronal BC1 and BC200 ribonucleoprotein particles. J. Mol. Biol..

[32] Tiedge H., Fremeau R.T., Weinstock P.H., Arancio O., Brosius J. (1991). Dendritic location of neural BC1 RNA. Proc. Natl. Acad. Sci. USA.

[33] Lin D., Pestova T.V., Hellen C.U., Tiedge H. (2008). Translational control by a small RNA: Dendritic BC1 RNA targets the eukaryotic initiation factor4A helicase mechanism. Mol. Cell. Biol..

[34] Zalfa F., Adinolfi S., Napoli I., Kühn-Hölsken E., Urlaub H., Achsel T., Pastore A., Bagni C. (2005). Fragile X mental retardation protein (FMRP) binds specifically to the brain cytoplasmic RNAs BC1/BC200 via a novel RNA-binding motif. J. Biol. Chem..

[35] Zhong J., Chuang S.C., Bianchi R., Zhao W., Lee H., Fenton A.A., Wong R.K., Tiedge H. (2009). BC1 regulation of metabotropic glutamate receptor-mediated neuronal excitability. J. Neurosci..

[36] Gitaí D.L., Fachin A.L., Mello S.S., Elias C.F., Bittencourt J.C., Leite J.P., Passos G.A., Garcia-Cairasco N., Paçó-Larson M.L. (2011). The non-coding RNA BC1 is down-regulated in the hippocampus of Wistar Audiogenic Rat (WAR) strain after audiogenic kindling. Brain Res..

[37] Zeng X., Zong W., Gao Q., Chen S., Chen L., Zeng G., Huang W., Li Z., Zeng C., Xie Y. (2018). The Expression Alteration of BC1 RNA and its Interaction with Eukaryotic Translation Initiation Factor eIF4A Post-Status Epilepticus. Neurochem. Res..

[38] Iughetti L., Lucaccioni L., Fugetto F., Predieri B., Berardi A., Ferrari F. (2018). Brain-derived neurotrophic factor and epilepsy: A systematic review. Neuropeptides.

[39] Modarresi F., Faghihi M.A., Lopez-Toledano M.A., Fatemi R.P., Magistri M., Brothers S.P., van der Brug M.P., Wahlestedt C. (2012). Inhibition of natural antisense transcripts in vivo results in gene-specific transcriptional upregulation. Nat. Biotechnol..

[40] Lipovich L., Dachet F., Cai J., Bagla S., Balan K., Jia H., Loeb J.A. (2012). Activity-dependent human brain coding/noncoding gene regulatory networks. Genetics.

[41] Escayg A., Goldin A.L. (2010). Sodium channel SCN1A and epilepsy: Mutations and mechanisms. Epilepsia.

[42] Hsiao J., Yuan T.Y., Tsai M.S., Lu C.Y., Lin Y.C., Lee M.L., Lin S.W., Chang F.C., Liu Pimentel H., Olive C. (2016). Upregulation of Haploinsufficient Gene Expression in the Brain by Targeting a Long Non-coding RNA Improves Seizure Phenotype in a Model of Dravet Syndrome. EBioMedicine.

[43] Dutra M.R.H., Feliciano R.D.S., Jacinto K.R., Gouveia T.L.F., Brigidio E., Morris M., Naffah-Mazzacoratti M.D.G., Silva J.A. (2018). Protective role of UCP2 in oxidative stress and apoptosis during the silent phase of an experimental model of epilepsy induced by pilocarpine. Oxid. Med. Cell. Longev..

[44] Chen N., Gao Y., Yan N., Liu C., Zhang J.G., Xing W.M., Kong D.M., Meng F.G. (2014). High-frequency stimulation of the hippocampus protects against seizure activity and hippocampal neuronal apoptosis induced by kainic acid administration in macaques. Neuroscience.

[45] Jiang X., Yan Y., Hu M., Chen X., Wang Y., Dai Y., Wu D., Wang Y., Zhuang Z., Xia H. (2016). Increased level of H19 long noncoding RNA promotes invasion, angiogenesis, and stemness of glioblastoma cells. J. Neurosurg..

[46] Han C.L., Liu Y.P., Zhao X.M., Wang K.L., Chen N., Hu W., Zhang J.G., Ge M., Meng F.G. (2017). Whole-transcriptome screening reveals the regulatory targets and functions of long non-coding RNA H19 in epileptic rats. Biophys. Res. Commun..

[47] Han C.L., Ge M., Liu Y.P., Zhao X.M., Wang K.L., Chen N., Hu W., Zhang J.G., Li L., Meng F.G. (2018). Long non-coding RNA H19 contributes to apoptosis of hippocampal neurons by inhibiting let-7b in a rat model of temporal lobe epilepsy. Cell. Death Dis..

[48] Han C.L., Ge M., Liu Y.P., Zhao X.M., Wang K.L., Chen N., Meng W.J., Hu W., Zhang J.G., Li L. (2018). LncRNA H19 contributes to hippocampal glial cell activation via JAK/STAT signaling in a rat model of temporal lobe epilepsy. J. Neuroinflammation.

[49] Chureau C., Chantalat S., Romito A., Galvani A., Duret L., Avner P., Rougeulle C. (2011). Ftx is a non-coding RNA which affects Xist expression and chromatin structure within the X-inactivation center Region. Hum. Mol. Genet..

[50] Zhang W., Bi Y., Li J., Peng F., Li H., Li C., Wang L., Ren F., Xie C., Wang P. (2017). Long noncoding RNA FTX is upregulated in gliomas and promotes proliferation and invasion of glioma cells by negatively regulating miR-342-3p. Lab. Investig..

[51] Liu F., Yuan J.H., Huang J.F., Yang F., Wang T.T., Ma J.Z., Zhang L., Zhou C.C., Wang F., Yu J. (2016). Long noncoding RNA FTX inhibits hepatocellular carcinoma proliferation and metastasis by binding MCM2 and miR-374a. Oncogene.

[52] Long B., Li N., Xu X.X., Li X.X., Xu X.J., Guo D., Zhang D., Wu Z.H., Zhang S.Y. (2018). Long noncoding RNA FTX regulates cardiomyocyte apoptosis by targeting miR-29b-1-5p and Bcl2l2. Biochem. Biophys. Res. Commun..

[53] Li X., Giri V., Cui Y., Yin M., Xian Z., Li J. (2019). LncRNA FTX inhibits hippocampal neuron apoptosis by regulating miR-21-5p/SOX7 axis in a rat model of temporal lobe epilepsy. Biochem. Biophys Res. Commun..

[54] Zheng J., Yi D., Liu Y., Wang M., Zhu Y., Shi H. (2017). Long noncoding RNA UCA1 regulates neural Stem cell differentiation by controlling miR1/Hes1 expression. Am. J. Transl. Res..

[55] Miller-Delaney S.F., Bryan K., Das S., McKiernan R.C., Bray I.M., Reynolds J.P., Gwinn R., Stallings R.L., Henshall D.C. (2015). Differential DNA methylation profiles of coding and non-coding genes define hippocampal sclerosis in human temporal lobe epilepsy. Brain.

[56] Wang H.K., Yan H., Wang K., Wang J. (2017). Dynamic regulation effect of long non-coding RNA-UCA1 on NF-kB in hippocampus of epilepsy rats. Eur. Rev. Med. Pharmacol. Sci..

[57] Wang W., Wu Y., Zhang G., Fang H., Wang H., Zang H., Xie T., Wang W. (2014). Activation of Nrf2-ARE signal pathway protects the brain from damage induced by epileptic seizure. Brain Res..

[58] Geng J.F., Liu X., Zhao H.B., Fan W.F., Geng J.J., Liu X.Z. (2018). LncRNA UCA1 inhibits epilepsy and seizure-induced brain injury by regulating miR-495/Nrf2-ARE signal pathway. Int. J. Biochem. Cell. Biol..

[59] Zhang X., Hamblin M.H., Yin K.J. (2017). The long noncoding RNA Malat1: Its physiological and pathophysiological functions. RNA Biol..

[60] Bernard D., Prasanth K.V., Tripathi V., Colasse S., Nakamura T., Xuan Z., Zhang M.Q., Sedel F., Jourdren L., Coulpier F. (2010). A long nuclear-retained non-coding RNA regulates Synaptogenesis by modulating gene expression. EMBO J..

[61] Grant S.G. (2012). Synaptopathies: Diseases of the synaptome. Curr. Opin. Neurobiol..

[62] Wu Q., Yi X. (2018). Down-regulation of Long Noncoding RNA MALAT1 Protects Hippocampal Neurons Against Excessive Autophagy and Apoptosis via the PI3K/Akt Signaling Pathway in Rats with Epilepsy. J. Mol. Neurosci..

[63] Xie N., Cao L., Zhao X., Jiang H., Chi Z. (2011). Diazoxide preconditioning against seizure-induced oxidative injury is via the PI3K/Akt pathway in epileptic rat. Neurosci. Lett..

[64] Lu D., Luo P., Wang Q., Ye Y., Wang B. (2017). LncRNA PVT1 in cancer: A review and meta-analysis. Clin. Chim. Acta.

[65] Zhao T., Ding Y., Li M., Zhou C., Lin W. (2019). Silencing lncRNA PVT1 inhibits activation of astrocytes and increases BDNF expression in hippocampus tissues of rats with epilepsy by downregulating the Wnt signaling pathway. J. Cell. Physiol..

[66] Mercer T.R., Qureshi I.A., Gokhan S., Dinger M.E., Li G., Mattick J.S., Mehler M.F. (2010). Long noncoding RNAs in neuronal-glial fate specification and oligodendrocyte lineage maturation. BMC Neurosci..

[67] Roberts T.C., Morris K.V., Wood M.J. (2014). The role of long non-coding RNAs in neurodevelopment, brain function and neurological disease. Philos. Trans. R. Soc. Lond. B. Biol. Sci..

[68] Feng J., Bi C., Clark B.S., Mady R., Shah P., Kohtz J.D. (2006). The Evf-2 noncoding RNA is transcribed from the Dlx-5/6 ultraconserved region and functions as a Dlx-2 transcriptional coactivator. Genes Dev..

[69] Bond A.M., Vangompel M.J., Sametsky E.A., Clark M.F., Savage J.C., Disterhoft J.F., Kohtz J.D. (2009). Balanced gene regulation by an embryonic brain ncRNA is critical for adult hippocampal GABA circuitry. Nat. Neurosci..

[70] Cajigas I., Chakraborty A., Swyter K.R., Luo H., Bastidas M., Nigro M., Morris E.R., Chen S., VanGompel M.J.W., Leib D. (2018). The Evf2 Ultraconserved Enhancer lncRNA Functionally and Spatially Organizes Megabase Distant Genes in the Developing Forebrain. Mol. Cell..

[71] An H., Williams N.G., Shelkovnikoava T.A. (2018). NEAT1 and paraspeckles in neurodegenerative diseases: A missing lnc found?. Noncoding RNA Res..

[72] Hempelmann A., Taylor K.P., Heils A., Lorenz S., Prud’homme J.F., Nabbout R., Dulac O., Rudolf G., Zara F., Bianchi A. (2006). Exploration of the genetic architecture of idiopathic generalized epilepsies. Epilepsia.

[73] Barry G., Briggs J.A., Hwang D.W., Nayler S.P., Fortuna P.R., Jonkhout N., Dachet F., Maag J.L., Mestdagh P., Singh E.M. (2017). The long non-coding RNA NEAT1 is responsive to neuronal activity and is associated with hyperexcitability states. Sci. Rep..

[74] Meng L., Ward A.J., Chun S., Bennett C.F., Beaudet A.L., Rigo F. (2015). Towards a therapy for Angelman syndrome by targeting a long non-coding RNA. Nature.

[75] Adams B.D., Parsons C., Walker L., Zhang W.C., Slack F.J. (2017). Targeting noncoding RNAs in Disease. J. Clin. Investig..

[76] Miller T.M., Pestronk A., David W., Rothstein J., Simpson E., Appel S.H., Andres P.L., Mahoney K., Allred P., Alexander K. (2013). An antisense oligonucleotide against SOD1 delivered intrathecally for patients with SOD1 familial amyotrophic lateral sclerosis: A phase 1, randomised, first-in-man study. Lancet Neurol..

[77] Chiriboga C.A., Swoboda K.J., Darras B.T., Iannaccone S.T., Montes J., De Vivo D.C., Norris D.A., Bennett C.F., Bishop K.M. (2016). Results from a phase 1 study of nusinersen (ISIS-SMN(Rx)) in children with spinal muscular atrophy. Neurology.

[78] Pan J.-J., Xie X.-J., Li X., Chen W. (2015). Long Non-coding RNAs and Drug Resistance. Asian Pac. J. Cancer Prev..

[79] Zhou X., Yin C., Dang Y., Ye F., Zhang G. (2015). Identification of the long non-coding RNA H19 in plasma as a novel biomarker for diagnosis of gastric cancer. Sci. Rep..

[80] Martignano F., Rossi L., Maugeri A., Gallà V., Conteduca V., De Giorgi U., Casadio V., Schepisi G. (2017). Urinary RNA-based biomarkers for prostate cancer detection. Clin. Chim. Acta.

[81] Akers J.C., Gonda D., Kim R., Carter B.S., Chen C.C. (2013). Biogenesis of extracellular vesicles (EV): Exosomes, microvesicles, retrovirus-like vesicles, and apoptotic bodies. J. Neurooncol..

[82] Sarfi M., Abbastabar M., Khalili E. (2019). Long noncoding RNAs biomarker-based cancer assessment. J. Cell. Physiol..

[83] Kogo R., Shimamura T., Mimori K., Kawahara K., Imoto S., Sudo T., Tanaka F., Shibata K., Suzuki A., Komune S. (2011). Long noncoding RNA HOTAIR regulates polycomb-dependent chromatin modification and is associated with poor prognosis in colorectal cancers. Cancer Res..

